# Histone methylation-mediated silencing of miR-139 enhances invasion of non-small-cell lung cancer

**DOI:** 10.1002/cam4.505

**Published:** 2015-08-08

**Authors:** Kousuke Watanabe, Yosuke Amano, Rie Ishikawa, Mitsuhiro Sunohara, Hidenori Kage, Junji Ichinose, Atsushi Sano, Jun Nakajima, Masashi Fukayama, Yutaka Yatomi, Takahide Nagase, Nobuya Ohishi, Daiya Takai

**Affiliations:** 1 Department of Respiratory Medicine, The University of Tokyo HospitalHongo, Bunkyo-ku, Tokyo, Japan; 2 Department of Cardiothoracic Surgery, The University of Tokyo HospitalHongo, Bunkyo-ku, Tokyo, Japan; 3 Department of Pathology, The University of Tokyo HospitalHongo, Bunkyo-ku, Tokyo, Japan; 4 Department of Clinical Laboratory, The University of Tokyo HospitalHongo, Bunkyo-ku, Tokyo, Japan

**Keywords:** Epigenetic repression, histones, lung neoplasms, microRNAs, neoplasm metastasis

## Abstract

MicroRNA expression is frequently altered in human cancers, and some microRNAs act as oncogenes or tumor suppressors. MiR-139-5p (denoted thereafter as miR-139) has recently been reported to function as a tumor suppressor in several types of human cancer (hepatocellular carcinoma, colorectal cancer, breast cancer, and gastric cancer), but its function in non-small-cell lung cancer (NSCLC) and the mechanism of its suppression have not been studied in detail. MiR-139 was suppressed frequently in primary NSCLCs. MiR-139 is located within the intron of *PDE2A* and its expression was significantly correlated with the expression of *PDE2A*. A chromatin immunoprecipitation assay revealed that miR-139 was epigenetically silenced by histone H3 lysine 27 trimethylation (H3K27me3) of its host gene *PDE2A* and this process was independent of promoter DNA methylation. Pharmacological inhibition of both histone methylation and deacetylation-induced miR-139 with its host gene *PDE2A*. Ectopic expression of miR-139 in lung cancer cell lines did not affect the proliferation nor the migration but significantly suppressed the invasion through the extracellular matrix. In primary NSCLCs, decreased expression of miR-139 was significantly associated with distant lymph node metastasis and histological invasiveness (lymphatic invasion and vascular invasion) on both univariate and multivariate analyses. Collectively, these results suggest that H3K27me3-mediated silencing of miR-139 enhances an invasive and metastatic phenotype of NSCLC.

## Introduction

MicroRNAs (miRNAs) are versatile regulators of gene expression. They negatively regulate thousands of target genes through mRNA destabilization and/or translational suppression 1. Through the posttranscriptional regulation of many target genes, miRNAs are involved in many biological processes, including cell cycle control, apoptosis, and differentiation.

MiRNA expression is frequently altered in human cancers both genetically and epigenetically 2–6. Some miRNAs function as oncogenes or tumor suppressors, and are involved in every step of carcinogenesis from initiation to acquisition of invasive and metastatic phenotypes 7.

Epigenetic silencing by DNA methylation is one of many mechanisms of miRNA suppression in human cancer 2,4–6. A recent genome-wide study of histone modifications in prostate cancer revealed that histone H3 lysine 27 trimethylation (H3K27me3) as a mechanism of tumor suppressor gene silencing in cancer that occurs independently of promoter DNA methylation 8. Therefore, H3K27me3 may play a role in silencing tumor-suppressive miRNAs in human cancer.

Non-small-cell lung cancer (NSCLC) is the most common histological subtype of lung cancer. Recent studies have demonstrated that some miRNAs have important roles in the metastatic process of lung cancer. For example, the miR-200 family 9 and miR-328 10 have been reported to function as antimetastatic miRNAs in NSCLC.

Recently, miR-139-5p (denoted thereafter as miR-139) has been shown to function as an antimetastatic miRNA in hepatocellular carcinoma 11 and breast cancer 12. MiR-139 has also been discovered to play an important role in the HER2-mediated metastatic process of gastric cancer 13. Ectopic expression of miR-139 suppresses the proliferation, migration, and invasion of esophageal and colorectal cancer 14,15. These studies suggest that the downregulation of miR-139 is a common feature of wide spectrum of human malignancies. The purpose of the present study was to analyze the function and mechanism of transcriptional regulation of miR-139 in NSCLC.

In the present study, miR-139 was frequently suppressed in lung cancer cell lines and primary NSCLCs. The chromatin immunoprecipitation (ChIP) assay was performed to analyze the transcriptional regulation of miR-139. We found that miR-139 was epigenetically silenced with its host gene *PDE2A* by H3K27me3 in lung cancer cells. Ectopic expression of miR-139 in lung cancer cell lines suppressed the invasion through the extracellular matrix. In primary NSCLCs, decreased expression of miR-139 was significantly associated with distant lymph node metastasis and histological invasiveness.

## Materials and Methods

### Lung cancer cell lines

Lung cancer cell lines were obtained from the American Type Culture Collection (Manassas, VA) and the Health Science Research Resources Bank (Osaka, Japan). Normal human bronchial epithelial cells (NHBE) were obtained from Lonza (Basel, Switzerland). These cell lines were cultured according to each manufacturer's protocol. Genomic DNA was extracted using the standard proteinase K/phenol method 16. Total RNA was isolated using RNAiso (Takara, Shiga, Japan).

### Samples of primary NSCLCs

Samples of primary NSCLCs were obtained from patients who had undergone surgical resection at the University of Tokyo Hospital. Informed consent was obtained from all the patients, and the Institutional Ethical Review Board approved the study. Total RNA was isolated as described above.

### Analysis of miRNA and host gene expression

MiRNA expression was determined using TaqMan microRNA assay (Applied Biosystems, Foster City, CA). U6 small nuclear RNA (RNU6B) was used as an internal control. For the analysis of *PDE2A* expression, total RNA was reverse-transcribed with SuperScript III (Invitrogen, Carlsbad, CA) and SYBR green RT-PCR was performed using THUNDERBIRD SYBR qPCR Mix (Toyobo, Osaka, Japan). Human brain total RNA (Ambion, Austin, TX) was used as a positive control for the RT-PCR of the long *PDE2A* transcript. PCR conditions and primer sequences are listed in Table S1.

### ChIP assays

ChIP analysis was performed using a OneDay ChIP Kit (Diagnode, Philadelphia, PA) as previously described 5,17. The antibodies used were histone H3 (tri methyl K4) antibody (39915, Lot number 31712004; Active Motif, Carlsbad, CA), histone H3 (tri methyl K9) antibody (39161, Lot number 13509002; Active Motif), and histone H3 (tri methyl K27) antibody (39155, Lot number 25812014; Active Motif). Immunoprecipitated DNA was quantified using SYBR green RT-PCR with THUNDERBIRD SYBR qPCR Mix (Toyobo). PCR conditions and the primer sequences are listed in Table S1.

### Analysis of DNA methylation

Genomic DNA was treated with sodium bisulfite according to a previously described protocol 5. Bisulfite PCR was performed using AmpliTaq Gold 360 Master Mix (Applied Biosystems). PCR conditions and the primer sequences are listed in Table S1.

### Copy number and mutation analysis

Alterations in the copy number of the miR-139 locus were determined using real-time PCR-based method 18. As a control, genomic DNA was extracted from the white blood cells of a healthy volunteer in our research team. Genomic DNA was digested with *Eco*RI (New England Biolabs, Ipswich, MA), purified using phenol/chloroform extraction, and used as a template for SYBR green RT-PCR with THUNDERBIRD SYBR qPCR Mix (Toyobo). *GAPDH* gene was used as an internal control. The PCR products of the miR-139 locus were sequenced to analyze the mutation status of miR-139. PCR conditions and the primer sequences are listed in Table S1.

### Epigenetic drug treatment

Cells were treated with 3-Deazaneplanocin A (DZNep, Cayman chemical, Ann Arbor, MI) for 48 h, Tricostatin A (TSA; Sigma-Aldrich, St Louis, MO) for 24 h, or their combination. Cells were first grown in growth medium containing DZNep, TSA, their combination, or Dimethyl sulfoxide (DMSO, control). After 24 h of incubation, the medium was replaced with fresh medium containing DZNep or DMSO and the cells were incubated for an additional 24 h. After 48-h treatment, total RNA was isolated using RNAiso (Takara). NCI-H2170 cells treated with DZNep (30 *μ*mol/L), TSA (0.1 *μ*mol/L), or their combination. ABC-1 cells treated with DZNep (30 *μ*mol/L), TSA (0.3 *μ*mol/L), or their combination.

### RNA interference

The siRNAs targeting *EZH2* (SASI_Hs01_00147882) and negative control (MISSION siRNA Universal Negative Control) were obtained from Sigma-Aldrich. Cells were transfected with 100 nmol/L final concentration of siRNA using N-TER Nanoparticle siRNA Transfection System (Sigma-Aldrich). After 9 h of incubation, the transfection medium was replaced with fresh medium containing TSA (0.1 *μ*mol/L for NCI-H2170 and 0.5 *μ*mol/L for ABC-1) or DMSO and the cells were incubated for an additional 24 h. After 24-h drug exposure, the cells were grown in drug-free medium for an additional 24 h, and the total RNA was isolated using RNAiso (Takara). The effect of siRNA was confirmed using SYBR green RT-PCR with THUNDERBIRD SYBR qPCR Mix (Toyobo). PCR conditions and the primer sequences are listed in Table S1.

### Overexpression of miRNA

The pol III-dependent miRNA expression vector was constructed as described previously 5,19. An empty vector (which contained five Ts after the U6 promoter) vector was used as a negative control. The DNA sequences of oligo DNAs used for the vector construction are listed in Table S1.

These vectors were transfected into NCI-H2170 and ABC-1 cells using HilyMax (Dojindo Laboratories, Kumamoto, Japan) followed by hygromycin selection (800 *μ*g/mL) for 1 week. After the selection, cells were grown in 270 *μ*g/mL hygromycin to maintain the hygromycin-resistant phenotype and used for subsequent analyses.

### Cell proliferation, migration and invasion assay

For cell proliferation assay, cells were plated in a six-well plate and triplicate wells were counted every other day. Transwell migration and invasion assays were performed using a 96 well basement membrane extract (BME) cell invasion assay kit (Trevigen, Gaithersburg, MD). For transwell invasion assay, chambers were coated with BME.

### Statistical analysis

The relationship between miRNA expression and clinicopathological findings were analyzed using Dr. SPSS II (SPSS, Chicago, IL).

## Results

### Suppression of miR-139 with its host gene *PDE2A* in primary NSCLCs and lung cancer cell lines

**Figure 1:**
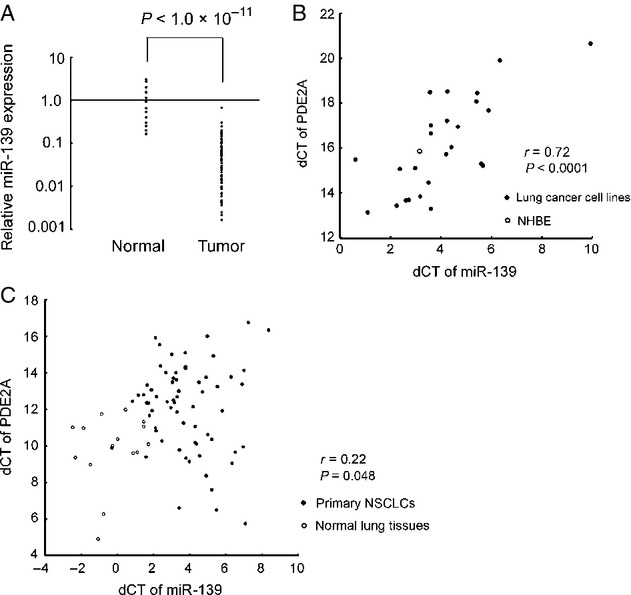
Expression of miR-139 in lung cancer. (A) Expression of miR-139 in primary NSCLCs (*n* = 75) and adjacent normal lung tissues (*n* = 15). The patient characteristics of the 75 primary NSCLCs are described in Table1A. Adjacent normal lung tissues were obtained from randomly selected 15 cases. The experiments were in duplicate and the expression levels are relative to the average expression of the 15 normal lung tissues. The statistical significance of the expression difference was determined using Student's *t*-test. (B) Association of miR-139 and *PDE2A* expression in 25 lung cancer cell lines and NHBE. U6 small nuclear RNA and *ACTB* was used as internal controls. The experiments were in triplicate and the list of cell lines is shown in Table S2. A Pearson's correlation coefficient was calculated between miR-139 and *PDE2A* expression. (C) Association of miR-139 and *PDE2A* expression in primary NSCLCs and adjacent normal lung tissues. U6 small nuclear RNA and *ACTB* was used as internal controls. A Pearson's correlation coefficient was calculated between miR-139 and *PDE2A* expression. NSCLCs, non-small-cell lung cancer; NHBE, normal human bronchial epithelial cells.

**Figure 2:**
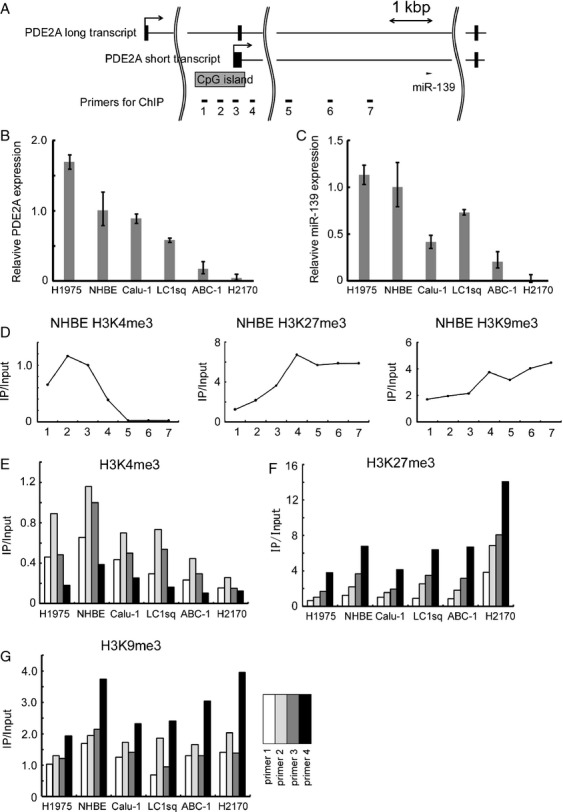
Chromatin immunoprecipitation assay. (A) Structure of *PDE2A* genomic locus. The locations of miR-139 and the ChIP primers are shown. (B) Expression of *PDE2A* short transcript in five lung cancer cell lines and NHBE. *ACTB* was used as an internal control. The data are shown as mean ± SD (*n* = 3). (C) Expression of miR-139 in five lung cancer cell lines and NHBE. U6 small nuclear RNA was used as an internal control. The data are shown as mean ± SD (*n* = 3). (D) Histone methylation status in NHBE. The horizontal axis indicates the numbers of primers shown in Figure2A. The experiments were in duplicate, and the results shown as the ratio of IP DNA to input DNA. IP/Input values were normalized to a control locus (*ACTB* promoter). (E–G) ChIP analysis of H3K4me3, H3K27me3, and H3K9me3 in five lung cancer cell lines and NHBE. The experiments were in duplicate, and IP/Input values were normalized to a control locus (*ACTB* promoter). ChIP, chromatin immunoprecipitation; NHBE, normal human bronchial epithelial cells.

In primary NSCLCs, miR-139 expression was significantly suppressed compared with adjacent normal lung tissue (*P* < 1.0 **×** 10^−11^, Fig.1A). On an average, a 14-fold expression difference in was detected between cancerous and noncancerous tissue. MiR-139 is located within the intron of its host gene *PDE2A* (Fig.2A). As intronic miRNAs are sometimes expressed with their host genes in a coordinated manner 20, we measured the expression of miR-139 and *PDE2A* in 25 lung cancer cell lines and NHBE cells (Fig.1B). The expression of miR-139 was significantly associated with the expression of *PDE2A* (Fig.1B). A mild but significant correlation was observed between miR-139 and *PDE2A* expression in primary NSCLCs and adjacent normal lung tissues (Fig.1C). These findings indicate that miR-139 is suppressed with its host gene *PDE2A* in NSCLC.

### Increased H3K27me3 and decreased H3K4me3 in lung cancer cell lines with low miR-139 and *PDE2A* expression

According to the UCSC Genome Bioinformatics Site (http://genome.ucsc.edu), *PDE2A* has transcriptional variants, and one short transcript has a CpG island at its 5′ end (Fig.2A). Expression of a long *PDE2A* transcript was detected in the brain, but not in the 25 lung cancer cell lines and NHBE cells even after 40 real-time PCR amplification cycles, suggesting that miR-139 is cosuppressed with the short *PDE2A* transcript in lung cancer cells.

To further analyze the transcriptional regulation of the short *PDE2A* transcript, we performed ChIP assay of NHBE cells and five lung cancer cell lines with various levels of *PDE2A* and *miR-139* expression (Fig.2B and C). We observed increased H3K4me3, a hallmark of active transcription, in the 5′ region of the short *PDE2A* transcript in NHBE cells (primer 2 in Fig.2D and E). This H3K4me3 peak was small in lung cancer cell lines with decreased expression of *PDE2A* (Fig.2E). A significant positive correlation was observed between *PDE2A* expression and H3K4me3 enrichment at primer 1 (Pearson's correlation coefficient *r* = 0.75, *P* = 0.04) or at primer 2 (*r* = 0.78, *P* = 0.03).

In addition to H3K4me3, two repressive histone modifications (H3K27me3 and H3K9me3) were analyzed. We found that H3K27me3 was enriched in the downstream region of the CpG island (primer 4 in Fig.2F). H3K27me3 was especially enriched in NCI-H2170 cells that had lowest *PDE2A* expression. A significant negative correlation was observed between *PDE2A* expression and H3K27me3 enrichment at primer 4 (*r* = −0.74, *P* = 0.04). No correlation was detected between *PDE2A* expression and H3K9me3 status (Fig.2G). The change in status of histone methylation was independent of the DNA methylation of the CpG island (Fig. S1A and B).

Finally, we analyzed the changes in copy number of the miR-139 locus to exclude the possibility of deletion of this chromosomal region (Fig. S1C). Neither significant loss in copy number nor mutations were detected at the miR-139 locus. Collectively, these findings suggested that miR-139 was epigenetically silenced with its host gene *PDE2A* by histone methylation and this process was independent of DNA methylation.

### Induction of miR-139 with its host gene *PDE2A* by the combination of DZNep and TSA

**Figure 3:**
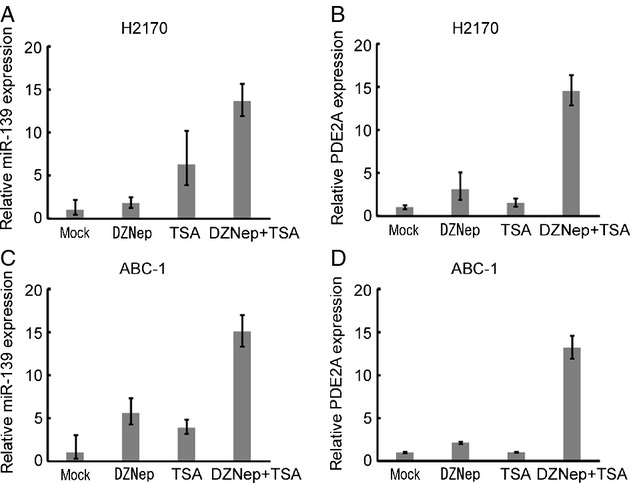
Induction of miR-139 and *PDE2A* by DZNep and TSA. (A and B) Expression of miR-139 and P*DE2A* of NCI-H2170 cells treated with DZNep (30 *μ*mol/L), TSA (0.1 *μ*mol/L), or their combination. The data are shown as mean ± SD (*n* = 3). (C and D) Expression of miR-139 and P*DE2A* of ABC-1 cells treated with DZNep (30 *μ*mol/L), TSA (0.3 *μ*mol/L), or their combination. The data are shown as mean ± SD (*n* = 3). DZNep, 3-Deazaneplanocin A; TSA, Tricostatin A.

It is reported that DZNep inhibits H3K27me3 in cancer cells 21. As H3K27me3 was especially enriched in NCI-H2170 cells that had lowest expression of *PDE2A* and miR-139 (Fig.2F), we tested whether DZNep, alone or in combination with histone deacetylation inhibitor TSA, can induce the expression of *PDE2A* and miR-139. As shown in Figure3A and B, DZNep alone did not largely affect the expression of *PDE2A* and miR-139, but the combination of DZNep and TSA significantly induced miR-139 expression together with its host gene *PDE2A*. Similar results were confirmed in ABC-1 cells (Fig.3C and D).

### Induction of miR-139 by the combination of TSA and *EZH2* knockdown

**Figure 4:**
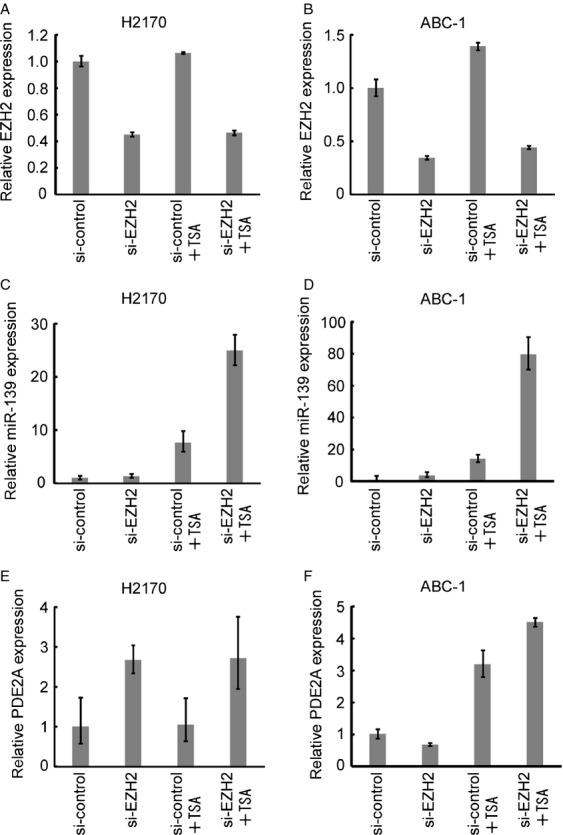
Effect of *EZH2* knockdown on miR-139 and *PDE2A* expression. (A and B) Expression of *EZH2* after *EZH2* knockdown with and without Tricostatin A (TSA) (0.1 *µ*mol/L for NCI-H2170 and 0.5 *µ*mol/L for ABC-1). The data are shown as mean ± SD (*n* = 3). (C and D) Expression of miR-139 after *EZH2* knockdown with and without TSA. The data are shown as mean ± SD (*n* = 3). (E and F) Expression of *PDE2A* after *EZH2* knockdown with and without TSA. The data are shown as mean ± SD (*n* = 3).

As EZH2 has histone methyltransferase activity with substrate specificity for H3K27 22, the effect of *EZH2* knockdown on miR-139 and *PDE2A* expression was analyzed in lung cancer cells. The combination of TSA and *EZH2* knockdown induced robust induction of miR-139 in NCI-H2170 and ABC-1 cells (Fig.4C and D). The expression of *PDE2A* was also induced by TSA and *EZH2* knockdown, although the degree of induction was smaller than that of miR-139 (Fig.4E and F).

### Suppression of invasion by miR-139 in lung cancer cells

**Figure 5:**
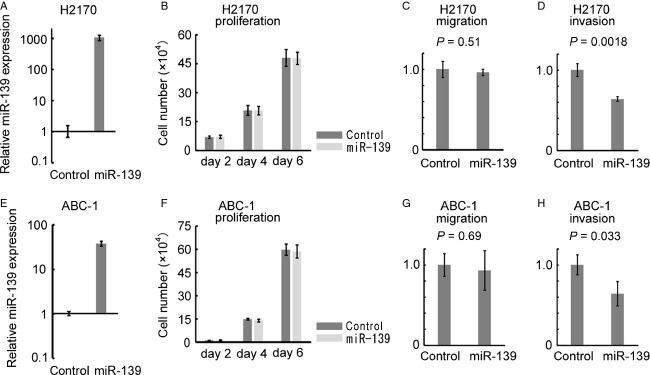
Overexpression of miR-139 in NCI-H2170 and ABC-1. (A and E) Stable overexpression of miR-139 in NCI-H2170 and ABC-1 cells. The vertical axis indicates miR-139 expression relative to the control vector. The data are shown as mean ± SD (*n* = 3). (B and F) Cell proliferation of NCI-H2170 and ABC-1 cells overexpressing miR-139. NCI-H2170 and ABC-1 cells were seeded at a density of 5 and 2 × 10^4^ cells per well in a 6-well plate on day 0, and triplicate wells were counted every other day. The data are shown as mean ± SD (*n* = 3). (C and G) Transwell migration assay of NCI-H2170 and ABC-1 cells overexpressing miR-139. The data are shown as mean ± SD (*n* = 3) and compared using Student's *t*-test. (D and H) Transwell invasion assay of NCI-H2170 and ABC-1 cells overexpressing miR-139. The data are shown as mean ± SD (*n* = 3) and compared using Student's *t*-test.

To evaluate the function of miR-139 in NSCLC, we overexpressed miR-139 in NCI-H2170 cells that has lowest miR-139 expression (Fig.5A). Overexpression of miR-139 using a U6 promoter-based expression vector 5,19 did not affect the proliferation (Fig.5B) nor the transwell cell migration (Fig.5C). However, miR-139 significantly suppressed the cell invasion through the extracellular matrix (Fig.5D). Similar results were confirmed in ABC-1 cells (Fig.5E–H). These results show that miR-139 has anti-invasive function in lung cancer cells.

### Association between miR-139 expression and invasive and metastatic phenotype in primary NSCLCs

We analyzed 75 primary NSCLCs from 75 histologically confirmed NSCLC patients. The clinical backgrounds of these patients are shown in Table1A. Forty patients did not have lymph node metastasis (N0 disease), 13 patients had local lymph node metastasis (N1 disease), and 22 patients had distant lymph node metastasis (N2 disease). Epidermal Growth Factor Receptor (EGFR) mutation status was available in 73 patients. We first noticed that patients with distant lymph node metastasis (N2 disease) had lower expression of miR-139 (Student's *t*-test *P* = 0.01) and we determined the cut-off value that best discriminate patients with distant lymph node metastasis from other patients. We divided the patients into two groups based on the miR-139 expression using this cut-off value. In a univariate analysis (chi-square test), low expression of miR-139 was significantly associated with distant lymph node metastasis (*P* = 0.038, Table1B). Moreover, low expression of miR-139 was significantly associated with the presence of both lymphatic and venous invasion (*P* = 0.022, Table1B). In contrast, miR-139 expression was independent of tumor size or EGFR mutation (Table1B). These results suggested that the loss of miR-139 expression (which was independent of oncogenic driver mutation) was associated not with the size of the primary tumor but with the metastatic and invasive features of lung cancer.

**Table 1:** miRNA expression and clinicopathological characteristics

	Number of cases (*n*)
(A) Clinical backgrounds of 75 cases	
Age	≥65 years, *n* = 41; <65 years, *n* = 34
Gender	Male, *n* = 50; female, *n* = 25
Smoking	Smoker, *n* = 49; nonsmoker, *n* = 25; unknown, *n* = 1
Histology	Adenocarcinoma, *n* = 61; squamous cell carcinoma, *n* = 11Adenosquamous carcinoma, *n* = 1; pleomorphic carcinoma, *n* = 2
Tumor size	T1, *n* = 35; T2–4, *n* = 40

	miR-139 low (*n*)	miR-139 high (*n*)	*P* value
(B) miRNA expression and clinicopathological characteristics (univariate analysis)			
Tumor size			
T1	22	13	0.84
T2–4	26	14	
Distant lymph node metastasis			
N0–1	30	23	0.038^*^
N2	18	4	
Lymphatic and venous invasion			
Both positive	17	3	0.022^*^
Others	31	24	
EGFR mutation			
Positive	16	9	0.89
Negative	30	18	

	Odds ratio (95% confidential interval)	*P* value
(C) miRNA expression and distant lymph node metastasis (multivariate analysis)		
miR-139 expression (low or high)	3.993 (1.1–14.488)	0.035^*^
Tumor size (T2–4 or T1)	6.629 (1.89–23.249)	0.003^*^

	Odds ratio (95% confidential interval)	*P* value
(D) miRNA expression and local invasiveness (multivariate analysis)		
miR-139 expression low or high)	5.415 (1.362–21.529)	0.016^*^
Smoking (smoker or nonsmoker)	4.749 (1.184–19.056)	0.028^*^

Asterisks (^*^) indicate statistical significance (p<0.05).

The association between miR-139 expression and distant lymph node metastasis or histological invasiveness was confirmed further using a multivariate analysis (stepwise logistic regression analysis). The explanatory variables used were age, gender, smoking, histology, tumor size, and miR-139 expression. In a multivariate analysis, miR-139 expression (*P* = 0.035, odds ratio [OR] = 3.993) and tumor size (*P* = 0.003, OR = 6.629) were associated with distant lymph node metastasis, whereas other clinical variables (age, gender, smoking status, and histology) were not associated with distant lymph node metastasis (Table1C). In addition, miR-139 expression (*P* = 0.016, OR = 5.415) and smoking (*P* = 0.028, OR = 4.749) were associated with the presence of both lymphatic and venous invasion, whereas other clinical variables (age, gender, histology, and tumor size) were not associated with the presence of both lymphatic and venous invasion (Table1D).

These observations showed that the loss of miR-139 expression was significantly associated with the invasive and metastatic phenotype of primary NSCLCs.

## Discussion

MiR-139 has been recently reported to function as an antimetastatic miRNA in hepatocellular carcinoma 11 and breast cancer 12. Downregulation of miR-139 has also been shown to play an important role in the HER2-mediated metastatic process of gastric cancer 13. In the present study, miR-139 was frequently suppressed in NSCLC, and overexpression of miR-139 suppressed invasion of lung cancer cells. This is the first report on the tumor suppressive function of this miRNA in lung cancer, and our results confirms that the downregulation of miR-139 plays an important role in metastatic process of various types of human cancer.

MiR-139 is located within the intron of *PDE2A*. Intronic miRNAs are sometimes expressed with their host genes in a coordinated manner 20. In a systematic analysis of expression of 175 miRNAs using various human tissues, miR-139 was one of miRNAs whose expression showed the strongest correlation with expression of its host gene 20. Our results also confirmed that miR-139 expression was correlated with expression of its host gene in lung cancer cell lines and primary NSCLCs. In primary NSCLC, the correlation was much weaker than that observed in cancer cell lines, probably due to the RNA degradation and the inclusion of stromal and inflammatory cells in clinical tumor samples. On the other hand, one study reported that miR-139 has its own transcriptional unit that is different from its host gene, and determined the miR-139 transcriptional start site (TSS) using gastric cancer cells by the rapid amplification of cDNA ends (RACE) assay 13. In NHBE and lung cancer cells, however, we did not detect a H3K4me3 peak in the TSS as determined by the RACE assay (primer 6 in Fig.2A). These results suggest that miR-139 is coregulated with *PDE2A* at least in lung cancer cells.

We showed here that the miR-139 was silenced with its host gene *PDE2A* by H3K27me3. H3K27me3 was enriched in the downstream region of the CpG island (primer 4 in Fig.2F). This finding is consistent with the recent genome-wide study showing that H3K27me3 is enriched in the proximal downstream region of TSSs 23. EZH2 has histone methyltransferase activity with substrate specificity for H3K27 22. We have also found that pharmacologic inhibition of both EZH2 and histone deacethylase (HDAC) induced *PDE2A* and miR-139 expression, which is consistent with the report that EZH2 require HDAC for its gene silencing activity 24. As Miranda et al. reports that DZNep globally inhibits histone methylation and is not necessarily specific to EZH2 25, the effect of *EZH2* knockdown on miR-139 and *PDE2A* expression was analyzed in two lung cancer cell lines. The combination of TSA and *EZH2* knockdown led to robust induction of miR-139, which confirms that H3K27me3 is involved in the silencing of miR-139 in lung cancer. The combination of TSA and *EZH2* knockdown also induced *PDE2A* expression, but the degree of induction was smaller than that observed in miR-139. This difference may be explained by the activation of a novel miR-139 promoter that is different from its host gene, or the secondary effect such as the induction of miRNAs that target *PDE2A*. Our results show that H3K27me3 plays a role in the regulation of miR-139 and *PDE2A* but other histone modifications may also be involved. Further research will fully elucidate the regulation of miR-139 in NSCLC.

*EZH2* overexpression is associated with a poor prognosis in lung cancer and the knockdown of EZH2 expression decreases the growth and invasion of lung cancer cells 26. Our results suggest that *EZH2* may exert its oncogenic function partially by silencing tumor-suppressive miRNAs, and further research is required to fully characterize the miRNAs silenced by H3K27me3 in NSCLC.

In primary NSCLCs, decreased expression of miR-139 was significantly associated with distant lymph node metastasis and histological invasiveness. Mascaux et al. analyzed the evolution of miRNA expression during human bronchial squamous carcinogenesis 27. They compared miRNA expression profiles of normal tissue, hyperplasia, metaplasia, dysplasia, in situ carcinoma, and invasive squamous cell carcinoma. Interestingly, miR-139 expression was preferentially lost in the final step of carcinogenesis: from in situ carcinoma to invasive squamous cell carcinoma. Our results suggest that miR-139 plays an important role in lung cancer metastasis not only in the very early stage of carcinogenesis, but also in the advanced stage of disease progression. MiR-139 reportedly target Rho-kinase 2 in hepatocellular carcinoma 11, *NR5A2* in esophageal cancer 14, *NOTCH1* and *IGF1R* in colorectal cancer 15,28. We think it would be interesting to study whether knockdown of miR-139 enhances invasive phenotype of lung cancer cells. Further investigation is required to elucidate the molecular mechanism which links miR-139 expression and an aggressive phenotype of lung cancer.

In conclusion, H3K27me3-mediated silencing of miR-139 may have an important role in lung cancer metastasis, and miR-139 expression is a promising biomarker of an invasive and metastatic phenotype of lung cancer.

## Supporting Information

**Figure S1.** Analysis of the DNA methylation status and the copy number changes. (A) Structure of *PDE2A* genomic locus. The locations of the CpG island and the bisulfite sequencing primers are shown. (B) Bisulfite sequencing of a CpG island. The open and filled circles represent unmethylated and methylated CpG sites, respectively. (C) Relative copy number of the miR-139 locus. The vertical axis indicates the relative copy number to NHBE cells. The data are shown as mean ± SD (*n* = 3). WBC, white blood cells.

**Table S1.** The list of primer sequences.

**Table S2.** Expression of miR-139 and *PDE2A* in 25 lung cancer cell lines and NHBE.
