# Supplementary figures and images for: Histone methylation-mediated silencing of miR-139 enhances invasion of non-small-cell lung cancer
Source: Cancer Med. 2015 Aug 8;4(10):1573–82. doi: 10.1002/cam4.505 (PMC4618627; doi:10.1002/cam4.505)

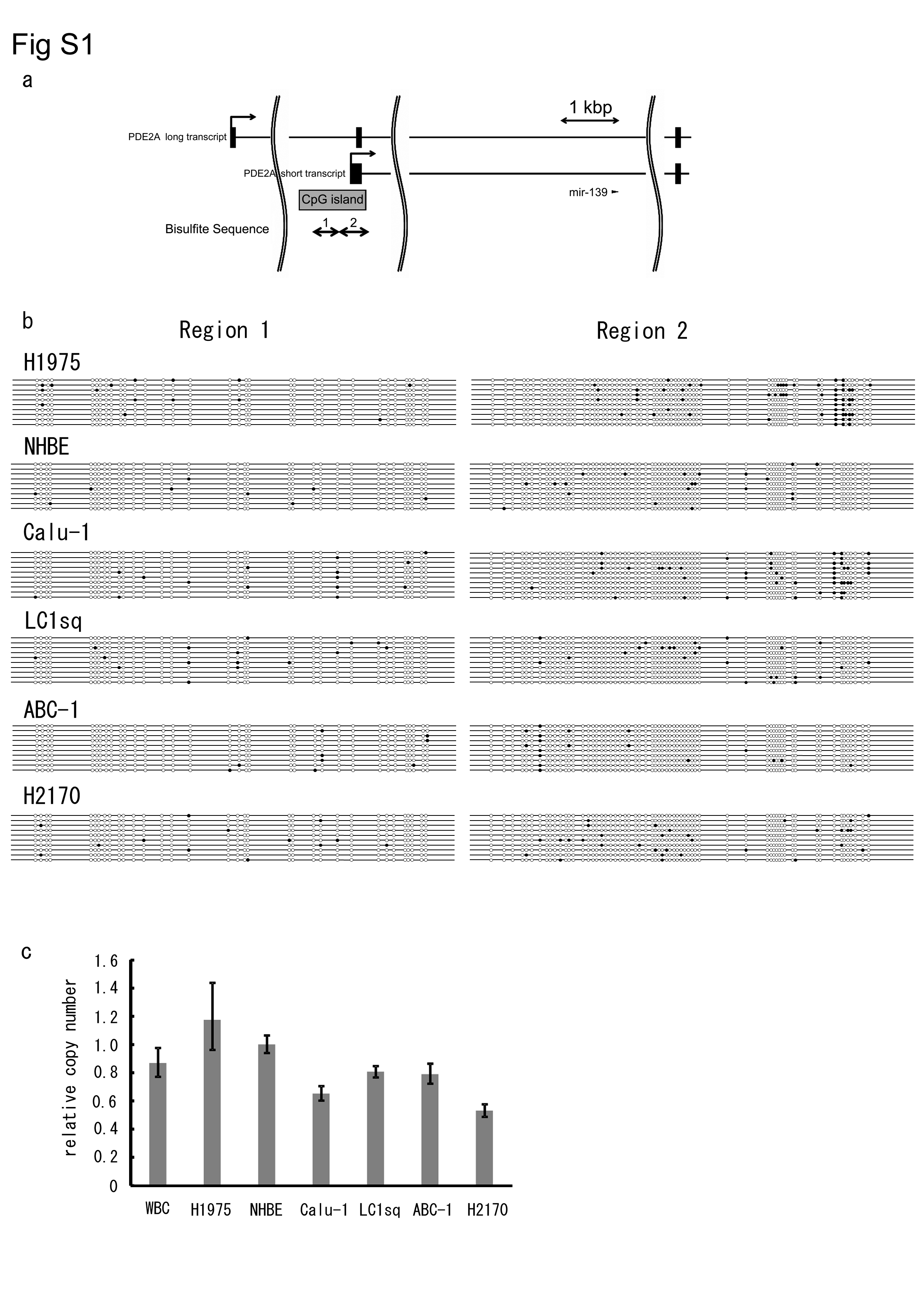

Supplement: Supplementary file 1 [file cam40004-1573-sd1.tif]
