# Supplementary material for: Histone methylation-mediated silencing of miR-139 enhances invasion of non-small-cell lung cancer
Source: Cancer Med. 2015 Aug 8;4(10):1573–82. doi: 10.1002/cam4.505 (PMC4618627; doi:10.1002/cam4.505)
Supplement: Supplementary file 2 [file cam40004-1573-sd2.doc]

**Supplementary Table 1 The list of primer sequences**

| Primer | | Sequence (5' to 3' direction) | Product size (bp) | Annealing Temp (ºC) | Other conditions |
| --- | --- | --- | --- | --- | --- |
| PDE2A long transcript detection | forward | GGCTGGGGAGGCGGACGATG |  |  |  |
|  | reverse | GCAGGCTGTCGGCGCATGG | 169 | 70 | two step, Betaine 1N |
| PDE2A RT-PCR | forward | CTGGGCTCTGTCATCGACATT |  |  |  |
|  | reverse | TGGGACTCACCATCCAGTAGGTAG | 113 | 60 |  |
| ACTB RT-PCR | forward | TTTGAGACCTTCAACACCCCA |  |  |  |
|  | reverse | TTTCGTGGATGCCACAGGA | 460 | 60 |  |
| EZH2 RT-PCR | forward | GATAGGTATTTTTGCCAAGA |  |  |  |
|  | reverse | AGAAGGCAATAAAAAGTTGAT | 291 | 55 |  |
| PDE2A ChIP | ChIP1 forward | GGTGGGCAGGTCTCTGTCGC |  |  |  |
|  | ChIP1 reverse | GCAGGCTCCTGAACCCAGAAGC | 139 | 55 | Betaine 1N |
|  | ChIP2 forward | GTCGCCACTTCTCTCGCATCT |  |  |  |
|  | ChIP2 reverse | CCACTGCGCCGGTCTCT | 132 | 55 | Betaine 1N |
|  | ChIP3 forward | CCGCCTGAGGAATTGGACAAC |  |  |  |
|  | ChIP3 reverse | GACACTCGGGCGGACCAG | 141 | 60 | Betaine 1N |
|  | ChIP4 forward | TCTAAGGAAAGGGAATTCGAG |  |  |  |
|  | ChIP4 reverse | GACTCTGAACTCCTTTCGGAT | 117 | 55 | Betaine 1N |
|  | ChIP5 forward | TCTTGTCCTCCCCCTATCACT |  |  |  |
|  | ChIP5 reverse | CATGTCTCCGCCAACTCTC | 148 | 60 | Betaine 1N |
|  | ChIP6 forward | CCTGACTGTCCCAGCGTGTT |  |  |  |
|  | ChIP6 reverse | GCGGGAGGCTGTGGGATAC | 118 | 60 | Betaine 1N |
|  | ChIP7 forward | TGTGCCCAGGACTACTGTACT |  |  |  |
|  | ChIP7 reverse | GGCTCCTTCTCTGAGACTGTA | 142 | 60 |  |
| ACTB ChIP | forward | CGTGCGCCGTTCCGAAAGT |  |  |  |
|  | reverse | CTTACCTGGCGGCGGGTGTG | 166 | 60 | Betaine 1N |
| Bisulfite PCR | Region 1 forward | GGTTTTTTTAGTTGGTTTGTTGTTAGT |  |  |  |
|  | Region 1 reverse | ATAACCCTAAAACCTACACCCTCTC | 470 | 55 |  |
|  | Region 2 forward | GTATAGGAGTAGAATTTAGAATTAGAGGAG |  |  |  |
|  | Region 2 reverse | TAACAACAAACCAACTAAAAAAACC | 527 | 55 |  |
| Copy Number | mir-139 forward | GACTCCGGCTTCAGTTGTTAC |  |  |  |
|  | mir-139 reverse | TTGGGAAGGGCGAGAG | 204 | 55 | Betaine 1N |
|  | GAPDH forward | CTCCTGGAAGGGCTTCGTAT |  |  |  |
|  | GAPDH reverse | GGGCCCAAGAGGTTGAAT | 127 | 55 | Betaine 1N |

| miRNA vector | Oligo miR-139 F | CACCTCTACAGTGCACGTGTCTCCAGTGTGGCTCGGAGGCTGGAGACGCGGCCCTGTTGGAGTTTTTGC |  |  |  |
| --- | --- | --- | --- | --- | --- |
|  | Oligo miR-139 R | GGCCGCAAAAACTCCAACAGGGCCGCGTCTCCAGCCTCCGAGCCACACTGGAGACACGTGCACTGTAGA |  |  |  |
